# Design of low density SNP chips for genotype imputation in layer chicken

**DOI:** 10.1186/s12863-018-0695-7

**Published:** 2018-12-04

**Authors:** Florian Herry, Frédéric Hérault, David Picard Druet, Amandine Varenne, Thierry Burlot, Pascale Le Roy, Sophie Allais

**Affiliations:** 1 NOVOGEN, 5 rue des Compagnons, Secteur du Vau Ballier, 22960 Plédran, France; 2 PEGASE, INRA, Agrocampus Ouest, 16 Le Clos, 35590 Saint-Gilles, France

**Keywords:** Imputation accuracy, Low density chip, Layer chickens, SNP density, Linkage disequilibrium, MAF, Degree of kinship

## Abstract

**Background:**

The main goal of selection is to achieve genetic gain for a population by choosing the best breeders among a set of selection candidates. Since 2013, the use of a high density genotyping chip (600K Affymetrix® Axiom® HD genotyping array) for chicken has enabled the implementation of genomic selection in layer and broiler breeding, but the genotyping costs remain high for a routine use on a large number of selection candidates. It has thus been deemed interesting to develop a low density genotyping chip that would induce lower costs. In this perspective, various simulation studies have been conducted to find the best way to select a set of SNPs for low density genotyping of two laying hen lines.

**Results:**

To design low density SNP chips, two methodologies, based on equidistance (EQ) or on linkage disequilibrium (LD) were compared. Imputation accuracy was assessed as the mean correlation between true and imputed genotypes. The results showed correlations more sensitive to false imputation of SNPs having low Minor Allele Frequency (MAF) when the EQ methodology was used. An increase in imputation accuracy was obtained when SNP density was increased, either through an increase in the number of selected windows on a chromosome or through the rise of the LD threshold. Moreover, the results varied depending on the type of chromosome (macro or micro-chromosome). The LD methodology enabled to optimize the number of SNPs, by reducing the SNP density on macro-chromosomes and by increasing it on micro-chromosomes. Imputation accuracy also increased when the size of the reference population was increased. Conversely, imputation accuracy decreased when the degree of kinship between reference and candidate populations was reduced. Finally, adding selection candidates’ dams in the reference population, in addition to their sire, enabled to get better imputation results.

**Conclusions:**

Whichever the SNP chip, the methodology, and the scenario studied, highly accurate imputations were obtained, with mean correlations higher than 0.83. The key point to achieve good imputation results is to take into account chicken lines’ LD when designing a low density SNP chip, and to include the candidates’ direct parents in the reference population.

## Background

In 2001, Meuwissen et al. [1] proposed a method known as “genomic selection”, consisting in using dense molecular markers such as single nucleotide polymorphisms (SNPs), to predict the genomic value of individuals without information regarding their phenotype. Since 2013, a high density (HD) genotyping SNP chip for chicken (600K Affymetrix® Axiom® HD genotyping array) [2] has enabled the implementation of genomic selection in layer and broiler breeding. When the genotypes and phenotypes of a reference population are known, it is possible to estimate the genomic value of a genotyped individual. The main objective is to choose, among the selection candidates of generation N, the best breeders for one or more traits. The selected breeders will then produce the individuals of generation N + 1.

However, the genotyping costs induced by the HD SNP chip remain high for a routine use on a large number of selection candidates. It has therefore been deemed interesting to develop a low cost genotyping approach, which can be achieved through the development of a low density genotyping chip. In that perspective, a set of SNP markers has to be selected to enable the imputation (prediction) of missing genotypes on a high density SNP chip. Imputation involves predicting the high density genotyping of selection candidates from their low density genotyping and from the high density genotyping of the reference population [3]. This approach relies on the Mendelian Laws of Inheritance and on linkage disequilibrium (LD).

To date, many studies on genotype imputation have been led in the bovine, porcine, ovine and poultry sectors. These studies have been conducted using different software such as FImpute [4], Beagle [5] or AlphaImpute [6]. Imputation accuracy can be calculated by comparing imputed genotype with true HD genotype, for each SNP.

Several factors influencing imputation accuracy have been studied in the literature. These factors need to be taken into account when designing a low density SNP chip, in order to get accurate imputation. The SNP density of low density SNP chips [7], the effect of linkage disequilibrium threshold [8], the effect of minor allele frequencies (MAF) of imputed SNPs [9, 10], the size of the reference population [11], and the degree of kinship between reference population and candidate population [8, 10] have all been identified in the literature as factors influencing imputation accuracy. These factors also have an impact on genomic evaluations [12–14]. However, the specificities of the *Gallus gallus* genome [15], especially with regard to the particular structure of the avian linkage disequilibrium [16–18] have not yet been fully investigated.

In this study, several factors affecting imputation accuracy were therefore investigated. These factors are: SNP density, LD threshold, MAF of imputed SNPs, chromosome size, the methodology used to design the low density SNP chip (based on physical equidistant intervals or on LD), the composition of the reference population in terms of size and degree of kinship, as well as the effect of using female genotypes. Various in silico analysis were conducted in order to choose the best strategy to achieve low density genotyping of two different laying hen lines.

## Methods

### Animals

**Fig. 1:**
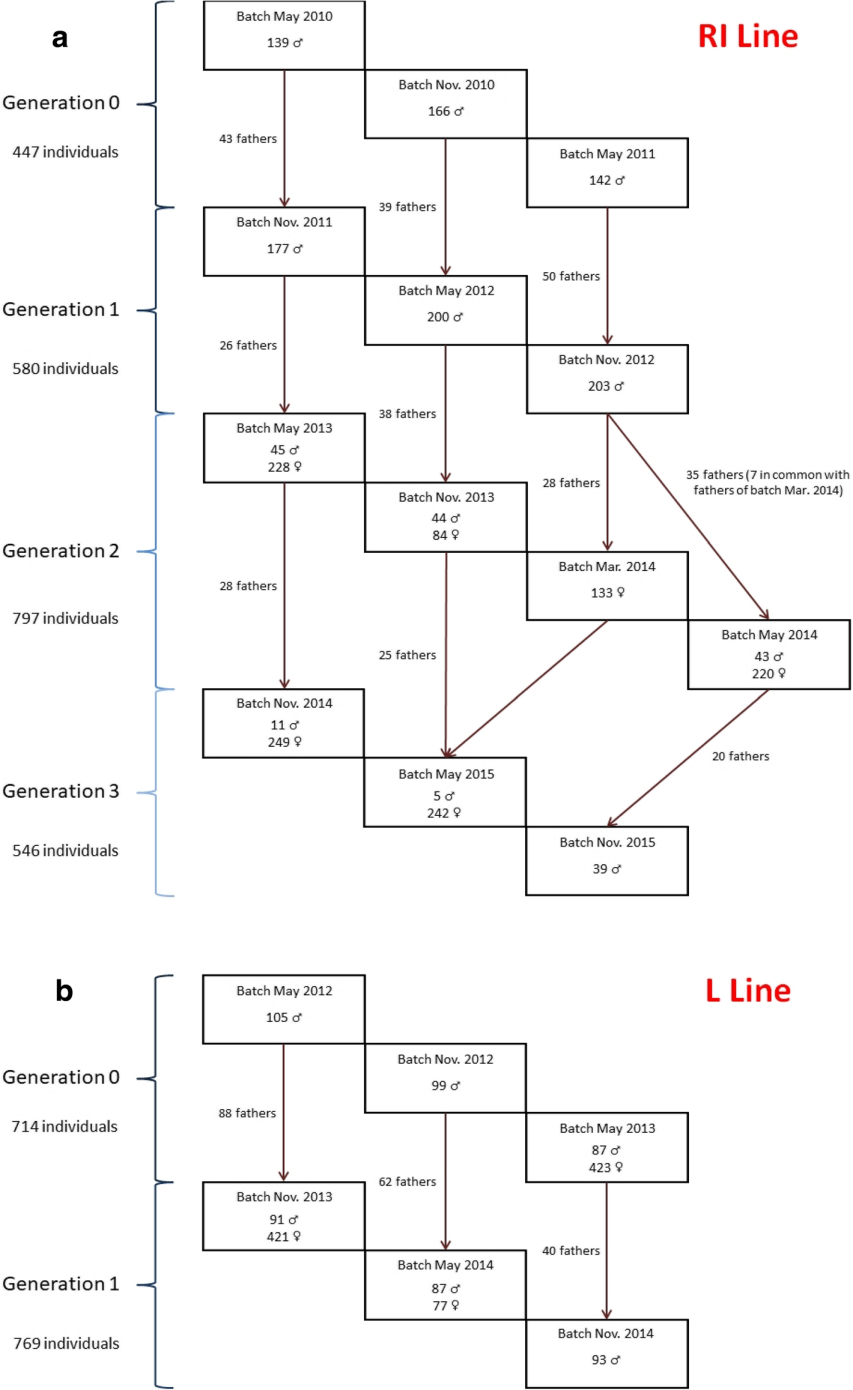
Population structure of the RI line (**a**) and L line (**b**) before quality control of genotyping

The populations studied were comprised of two different commercial pure lines of Rhode Island (RI) and Leghorn (L) laying hens. Each line was created and selected by Novogen (Plédran, France). The RI line was comprised of 2370 chickens split in four generations. The L line was comprised of 1483 chickens split in two generations. For both lines, each generation was divided in three batches and a new batch was produced every 6 months from 2010 to 2015 (Fig. 1). The selection objectives were the same for each batch and for each line, and remained the same over time. Animals were firstly selected on bird weight and egg quality, and secondly on egg quality and egg production. In addition, for each batch, the theoretical selection numbers defined were 50 males and 200 females and each sire was mated with 4 females.

For the RI line and for each batch, all male breeders were genotyped. Female breeders were genotyped as well, starting at the third generation (G2). From generation G3, born in November 2014, genomic selection was routinely implemented, by genotyping only the breeders. This enabled to reduce the generation interval for sires from 90 to 30 weeks. This accounts for the lower number of sires genotyped in November 2014 and May 2015.

For the L line and for each batch, all male breeders were genotyped. Female breeders were genotyped starting from the last batch of the first generation.

### Genotyping

Blood was taken from the brachial veins of the animals. DNA was extracted and hybridized using the 600K Affymetrix® Axiom® HD genotyping array [2]. Genotyping was performed by Ark-Genomics (Edinburgh, UK) for the first two generations of the RI line, and by the high-throughput genotyping platform Gentyane (Clermont-Ferrand, France), for the rest. Both platforms used Axiom Analysis Suite to create genotype files, which were received in PED format [19]. In order to check genotype consistency, PLINK identified 870 SNPs with visible strand inversion. Genotype harmonizer [20] identified 148 SNPs with invisible strand inversion. PLINK used both lists to flip the strand for the SNPs with strand inversion. Finally, the different sets of genotypes were successfully merged with PLINK.

Each individual was genotyped for 580,961 SNPs. According to the fifth annotation release of *Gallus gallus* genome [21], these SNPs were distributed over macro-chromosomes (1 to 5), intermediate chromosomes (6 to 10), micro-chromosomes (11 to 28 and 33), one linkage group (LGE64), two sexual chromosomes Z and W, as well as a group of 3724 SNPs with unknown location.

Genotypes were filtered through six successive steps with classical thresholds (Table 1), including individual call rate (> 95%), MAF (< 0.05), SNP call rate (> 95%), and Hardy-Weinberg equilibrium (*P* < 10^− 4^). Subsequently, the SNPs with unknown location or located on sexual chromosome W were removed, as well as the animals showing pedigree incompatibilities. Most of the SNPs had to be removed because they showed zero MAF. This was to be expected, since the HD SNP chip was designed for both layers and broilers and for a ratio of 1:2.

**Table 1:** Summary of the different steps of quality control

Genotypes filtration	RI Line	L Line
Individual Call Rate (> 95%)	8	3
MAF (= 0)	204,122	228,452
MAF (0 < X < 0.05)	54,650	99,000
SNP Call Rate (> 95%)	7541	2530
Hardy-Weinberg equilibrium (*P* < 10^−4^)	12,538	3857
SNP with unknown location or on W	1759	1453
Pedigree Incompatibility issues	0	6
**SNP retained for analyses**	**300,351**	**245,669**
**Animals retained for analyses**	**2362**	**1474**

In bold are the total number of SNPs and animals retained for analyses

For the RI line and for the L line respectively, 300,351 SNPs and 2362 individuals and 245,669 SNPs and 1474 individuals remained available for the analyses.

### Low density SNP chips design

From the remaining SNPs of the RI and L lines, several in silico low density SNP chips were designed by selecting a subset of SNPs (Table 2). Both lines were studied independently of each other. Low density genotyping was generated by masking all markers, except those corresponding to the in silico selected SNP panel for each line.

**Table 2:** Summary of the different low density SNP chips simulated

Methodology	SNP Chip	Number of SNP	
		RI Line	L Line
Equidistance	50Kequi	49,636	50,307
	40Kequi	40,160	39,838
	30Kequi	29,970	30,075
	20Kequi	19,910	19,948
	15Kequi	14,963	14,955
	**10Kequi**	**10,001**	**9966**
	7.5Kequi	7527	7496
	5Kequi	4991	4996
	4Kequi	4023	4000
	3Kequi	2992	3003
	2Kequi	2013	2003
Linkage Disequilibrium	LD0.8	21,717	18,052
	LD0.7	16,615	13,696
	**LD0.6**	13,214	**10,736**
	**LD0.5**	**10,711**	8626
	LD0.4	8521	6944
	LD0.3	6875	5578
	LD0.2	5371	4330
	LD0.1	3935	3232
	LD0.05	3205	2624

SNP chips in bold are SNP chips having an equivalent SNP density of 10 K SNPs

Many studies, mainly in non-avian sectors [7–9, 11, 22], focused on low density SNP chips designed according to the equidistance methodology (EQ), by choosing SNPs at regular physical intervals (in pb) along chromosomes. This methodology was therefore chosen. More precisely, for each interval, the SNP with the highest MAF, or the one located furthest on the left, in case of equivalent MAF, was chosen as representative of the interval. This way, 11 low density “equi” SNP chips designed according to this method were studied for each line, with SNP density ranging from 2K to 50K SNPs.

However, considering the heterogeneous structure of chicken linkage disequilibrium (LD) [18], it would have been better to design the low density SNP chips according to the intra-chromosomes LD, i.e. choosing tag SNPs at regular genetical intervals [23]. The low density SNP chips were therefore designed using the SS4I software [24]. This method made it possible to get clusters of SNPs according to a chosen LD threshold. For each cluster, the SNP with the highest MAF was then kept and used as representative of this cluster. Nine “LD SNP” chips designed with this method were studied, with LD threshold ranging from 0.05 to 0.8.

### Population scenarios

Eight population scenarios were set up and differed depending on the reference and candidate populations (Table 3). The individuals with the simulated low density genotyping were called “candidate population”. The reference population, in this study, refers to the individuals having high density genotyping and used to impute the candidates.

**Table 3:** Summary of the different scenarios depending on reference and candidate populations

	A	B	C	D_1_	D_2_	E	F	G	H	I
Reference Population	G0	G1	G0 + G1	G2(♂)	G2(♂ + ♀)	G1 + G2(♂)	G0 + G1 + G2(♂)	G0	G1(♂)	G0(♂)
Number of individuals	447	580	1027	73	735	653	1100	447	120	132
Selection Candidates	G1	G2	G2	G3	G3	G3	G3	G2	G3	G3
Number of individuals	580	794	794	541	541	541	541	794	541	541

♂ indicates that only male breeders are used in the reference population

♂ + **♀** indicates that both male and female breeders are used in the reference population

Scenarios (A), (B), (D_1_) and (D_2_) correspond to cases where the individuals of the candidate population are directly related to the reference population, because of the presence of their sires and/or dams in the reference population. In scenarios (C), (E) and (F), the individuals of the candidate population are also directly related to the reference population, but this time the size of the reference population was increased by adding individuals from previous generations. Finally, scenarios (G), (H) and (I) correspond to cases where the reference population does not include the sires of the candidate population, as a generation gap was introduced between the reference population and the candidate population.

The scenario (A) concerned RI and L lines, and the others concerned only the RI line.

### Imputation accuracy studies

Based on the low density SNP chips designed and on the population scenarios simulated, seven different parameters were studied, in order to investigate their influence on imputation accuracy.

The first four parameters were studied on scenario (A), for both lines, and concerned the low density SNP chips used. The lines were studied independently of each other.

1. The first parameter studied was the effect of SNP density on the low density SNP chips. This study was conducted with the 11 low density SNP chips designed with the EQ methodology as well as with the 9 low density SNP chips designed with the LD methodology.
2. Secondly, the effect of the LD threshold used to design the 9 low density SNP chips was investigated.
3. Thirdly, the effect of minor allele frequencies of imputed SNP on imputation accuracy was studied. This study was done using the low density SNP chips of 3K and 10K SNPs, designed according to the two methodologies, i.e. EQ and LD.
4. Fourth, the effect of the type of chromosome (micro, intermediate, macro or Z) was studied for both the equi and the LD chips, with a density of 3K and 10K SNPs.

The remaining last three parameters were studied at an equivalent SNP density of 10K SNPs, i.e. 10Kequi and LD0.5 low density SNP chips, and of 3K SNPs, i.e. 3Kequi and LD0.05 low density SNP chips. This was meant to focus on the effects of population structure and was done for the RI line only. The number of generations for the L line was insufficient and so did not enable to study the effects of population structure.

5) The effect of the size of reference population on imputation accuracy was studied by comparing scenarios (B) - (C) and scenarios (D) - (E) - (F), adding individuals from previous generations in the reference population in some of the scenarios.
6) The study of the effect of the degree of kinship between reference population and candidate population was conducted by comparing scenarios (B) - (C) - (G) and scenarios (D) - (F) - (H) - (I).
7) Finally, the effect of the presence or absence of the dams in the reference population was investigated on scenario (D), by taking into account or not taking into account the dams in the reference population.

### Software

FImpute V2.2 [4] was used to impute the high density genotyping of the selection candidates. FImpute is a software which was developed for livestock species and which uses pedigree information. It relies on overlapping sliding windows methodology to achieve imputations. Others imputation software like Beagle or AlphaImpute, which have also been reported in the literature, were tested on scenario (A), together with FImpute. However, given the relatively long execution time of Beagle (half a day) and AlphaImpute (1 week), in comparison to FImpute, only FImpute was subsequently used.

Moreover, because only the sires were present in the reference population, FImpute was used with the option *“turnoff_fam”* activated. This option enabled the software to turn off family imputation and to use the whole range of haplotypes of the reference population to achieve imputation. The information brought by the sire was not used. There was however one exception, which concerned scenario D_2_, where both sires and dams were present in G2. In that case, the analysis was carried out without activating the *“turnoff_fam”* option.

### Imputation accuracy

Following the suggestion of Hickey et al. [25] and Calus et al. [26], imputation accuracy was assessed as the mean correlation between true and imputed genotypes. Indeed, for one SNP, the correlation was not dependent on MAF and could be used to assess imputation accuracy, rather than using genotype and allelic imputation error rates. Correlations were calculated one SNP at a time for all the candidates, as suggested in Pearson’s method. Mean correlation was then estimated on 300,351 correlations for the RI line, and on 245,669 correlations for the L line. The mean correlations obtained were subsequently compared for the different low density SNP chips and/or scenarios, using Student tests with type 1 error rate of 0.1%.

## Results

The influence of the parameters, i.e. marker density, LD threshold, MAF of imputed SNPs, chromosome type, and composition of the reference population through the cumulative use of generations in the reference population, degree of kinship between reference population and candidate population and the effect of using dams’ genotypes in the reference population, was investigated.

### Influence of marker density

**Fig. 2:**
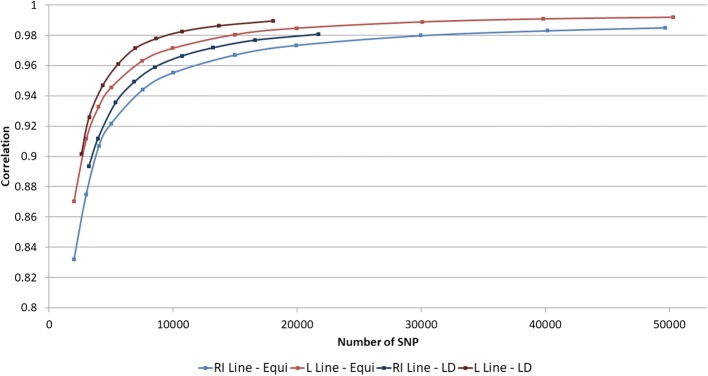
Evolution of mean correlations between true and imputed genotypes according to the number of SNPs on the low density SNP chips for both methodologies and both lines

The evolution of mean correlations between true and imputed genotypes according to the number of SNPs on the low density SNP chips was studied on scenario (A), for both lines and both methodologies (Fig. 2).

For both lines and for both methodologies, an increase in mean correlations was observed when the number of SNPs on the low density SNP chips was increased. Regarding the RI line and the EQ methodology, the mean correlation was 0.875 for 2992 SNPs and 0.973 for 19,910 SNPs. Regarding the same line and the LD methodology, the mean correlation was 0.893 for 3205 SNPs and 0.977 for 16,615 SNPs. An inflexion point was also noticed between 5000 SNPs and 10,000 SNPs.

**Fig. 3:**
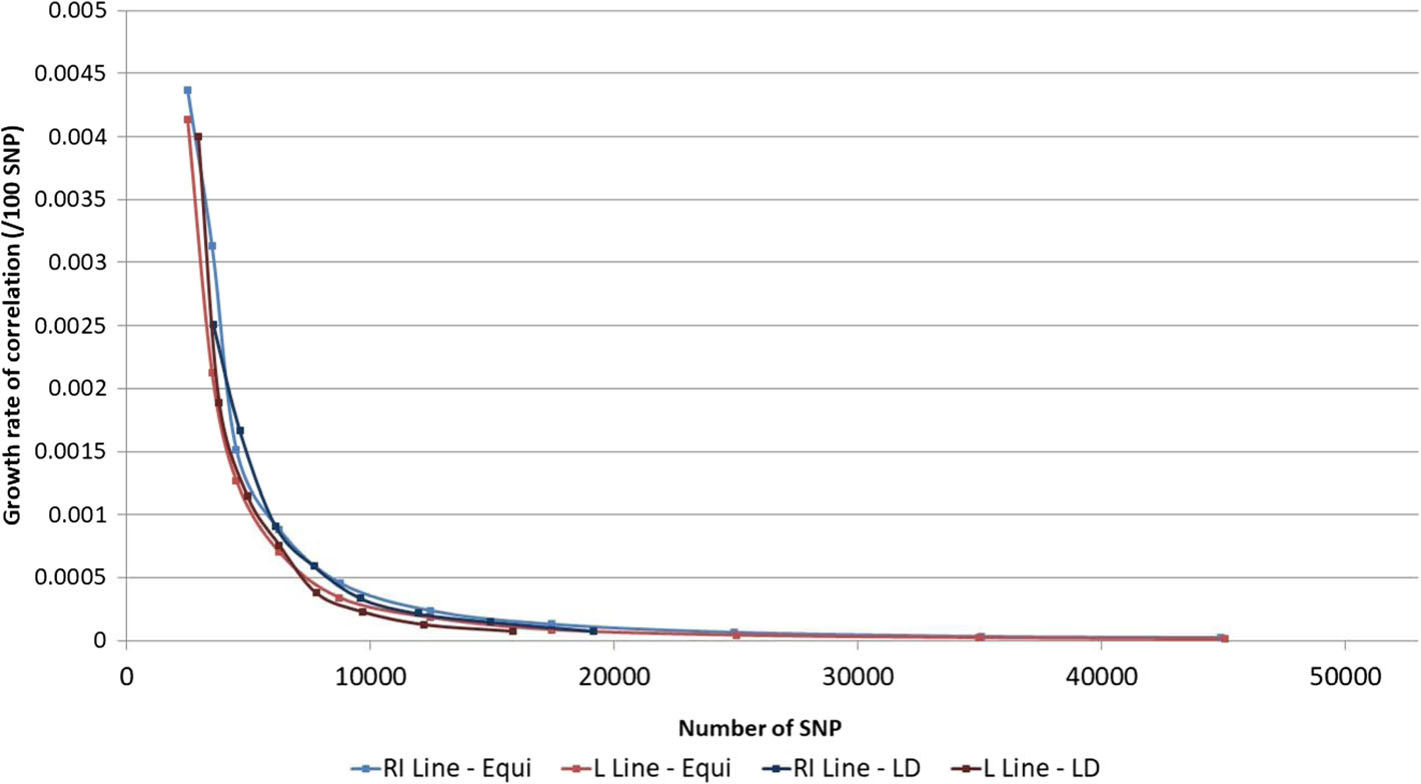
Evolution of the growth rate of mean correlations (/100 SNP) according to the number of SNPs on low density SNP chips for both methodologies and both lines

In addition, for both methodologies, the growth rate of the mean correlation was 0.004 for 3000 SNPs, which means that adding 100 SNPs on a low density SNP chip of 3000 SNPs would significantly increase the mean correlation of 0.004. Furthermore, the growth rate of the mean correlation was 7.0*10^− 5^ for 20,000 SNPs (Fig. 3), which was not significant.

Finally, given the fact that the inflexion point was between 5000 and 10,000 SNPs, a density of 10K SNPs enabled to reach steady and good imputation accuracy and was subsequently used throughout the rest of the present study. A density of 3K SNPs was also considered, in order to investigate the consequences that would result from a deteriorated, but nonetheless correct, imputation accuracy with mean correlations above 0.870.

### Influence of LD threshold

**Fig. 4:**
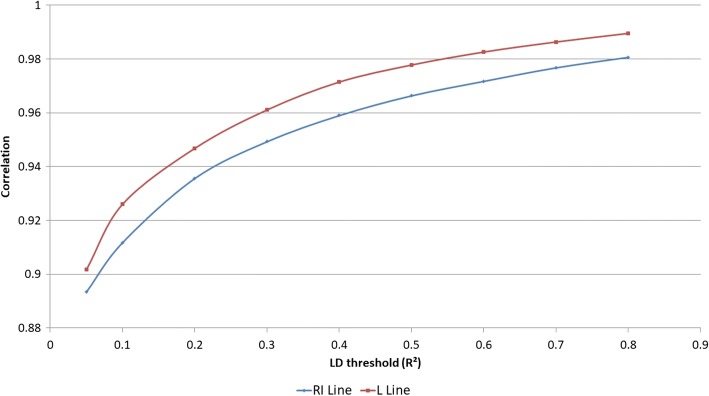
Evolution of mean correlations of true and imputed genotypes according to the LD threshold used to design the low density SNP chips, for both lines

The evolution of imputation accuracy as a function of LD threshold was studied on scenario (A) for both lines (Fig. 4). When the LD threshold used for the selection of representative SNP was increased, an increase in imputation accuracy was observed. For the RI line, the LD thresholds of 0.05, 0.5 and 0.8, respectively resulted in mean correlations of 0.893, 0.966 and 0.981. For the L line, the mean correlations were respectively 0.902, 0.978 and 0.990.

### Influence of MAF of imputed SNPs

**Fig. 5:**
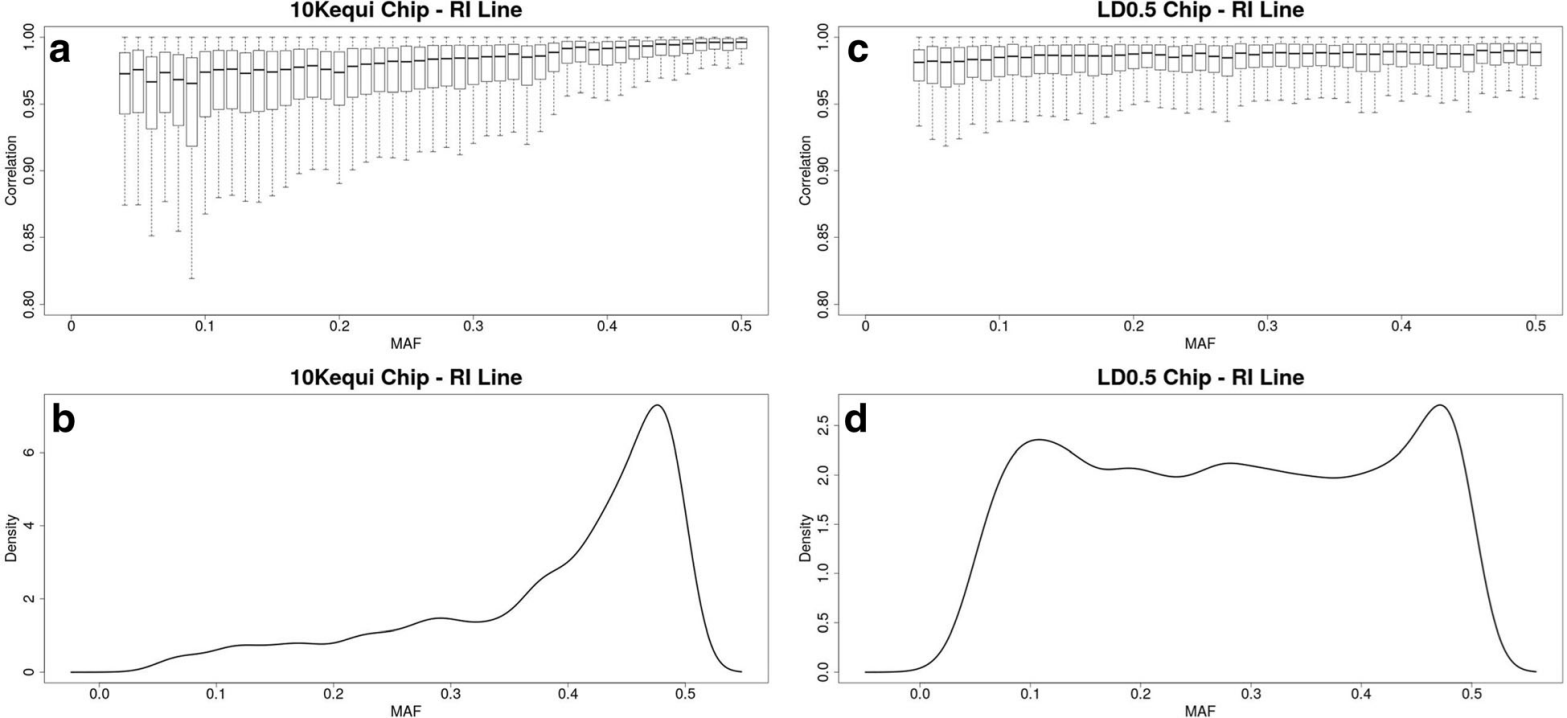
For the RI line, evolution of the correlations between true and imputed genotypes according to the MAF of imputed SNPs on the 10Kequi (**a**) and LD0.5 (**c**) SNP chips and distribution of the SNPs on the 10Kequi (**b**) and LD0.5 (**d**) SNP chips according to MAF

The influence of minor allele frequencies of imputed SNPs was studied on scenario (A), using both methodologies. The 10Kequi and the LD0.5 SNP chips were used for the RI line and the 10Kequi and the LD0.6 SNP chips were used for the L line. The influence of minor allele frequencies was also studied for a density of 3K SNPs, using the 3Kequi and LD0.05 SNP chips for the RI line (LD0.1 for the L line). The results for the density of 10K SNPs are the only ones shown, since they are similar to those obtained with the density of 3K SNPs. The same results were obtained for both lines and the results for the RI line are illustrated in Fig. 5.

With the 10Kequi SNP chip, an increase in imputation accuracy was noticed when the MAF of imputed SNPs was increased (Fig. 5a). Comparatively, more steady correlations were observed when MAF was increased using the LD methodology (Fig. 5c). Moreover, mean correlations were higher with the LD SNP chip than they were with the 10Kequi SNP chip. The variability of mean correlations according to MAF was also higher with the 10Kequi SNP chip than with the LD SNP chip.

Finally, by looking the MAF distribution of the SNPs of the different low density SNP chips, the SNPs of the 10Kequi SNP chip had mostly high MAF (Fig. 5b), whereas the SNPs of the LD0.5 SNP chip had both low and high MAF (Fig. 5d).

### Influence of the type of chromosome

The influence of the type of chromosome was studied on scenario (A), for both lines, with the two low density 10K SNPs, i.e. 10Kequi and LD0.5 chips for the RI line (LD0.6 for the L line). The influence of the type of chromosome was also studied at a density of 3K SNPs, using the 3Kequi and LD0.05 chips, for the RI line (LD0.1 for the L line). The results for the density of 10K SNPs are the only ones shown, since they are similar to those obtained with the density of 3K SNPs. Chromosomes were split into four different groups: macro-chromosomes (1 to 5), intermediate chromosomes (6 to 10), micro-chromosomes (11 to 28 and 33), and sexual chromosome Z [15].

**Fig. 6:**
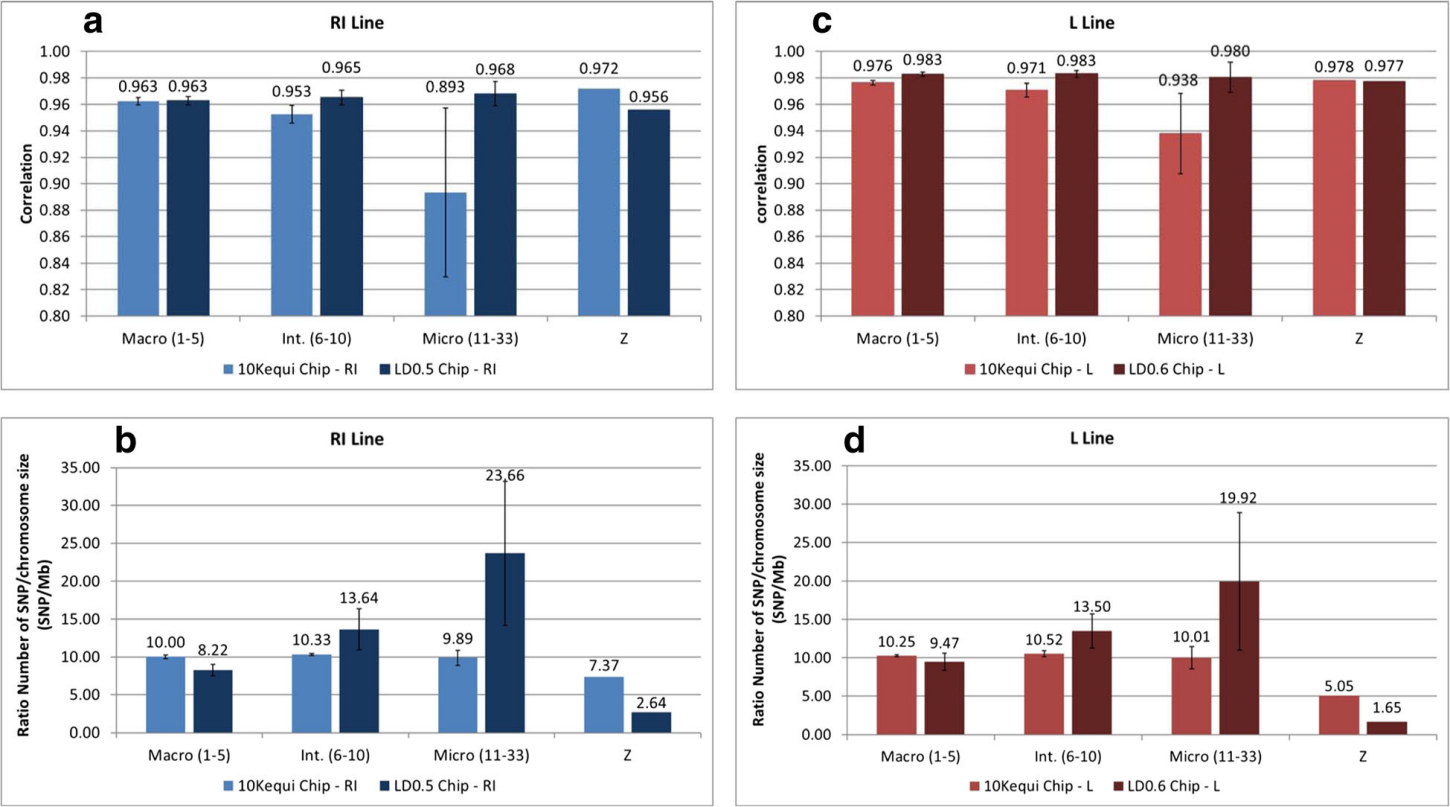
Evolution of mean correlations between true and imputed genotypes depending on the type of chromosome, for the RI line (**a**) and the L line (**c**), and evolution of the ratio number of SNP/Size of chromosome (SNP.Mb^− 1^) depending on the type of chromosome, for the RI line (**b**) and the L line (**d**)

Regarding the RI line (Fig. 6a) in conjunction with the EQ methodology (10Kequi SNP chip), mean correlations varied depending on the type of chromosome. When using the 10Kequi SNP chip, the mean correlations were 0.963 for macro-chromosomes, 0.953 for intermediate chromosomes, and 0.893 for micro-chromosomes. The differences in mean correlation were significant. As regards the LD0.5 SNP chip, the mean correlations were 0.963 for macro-chromosomes, 0.965 for intermediate chromosomes and 0.968 for micro-chromosomes. These differences in mean correlation were also significant and, except for macro-chromosomes, the differences between the 10Kequi SNP chip and the LD0.5 SNP chip were significant. As far as the LD0.5 SNP chip is concerned, imputation accuracy was rather steady regardless of the type of chromosome. Another point is that the standard error varied greatly depending on the type of chromosome, when the 10Kequi chip was used. This was not the case with the LD0.5 SNP chip, which showed stable and low variance for all types of chromosome. Finally, the results for sexual chromosome Z proved better with the EQ methodology than with the LD methodology. As far as the L line is concerned, similar observations were made regarding the EQ and LD methodologies, except for sexual chromosome Z (Fig. 6c).

To understand the performances of each low density SNP chip, and in turn to understand the evolution of mean correlations on each low density SNP chip, the ratio of selected SNPs on the low density SNP chips, per chromosome size (SNP.Mb^− 1^), was studied for each type of chromosome. For the RI line (Fig. 6b) in conjunction with the 10Kequi SNP chip, the ratios were steady regardless of the type of chromosome, with a ratio of 10.00 ± 0.23 SNP.Mb^− 1^ for macro-chromosomes, 10.33 ± 0.13 SNP.Mb^− 1^ for intermediate chromosomes and 9.89 ± 1.00 SNP.Mb^− 1^ for micro-chromosomes. For the L line (Fig. 6d) in conjunction with the 10Kequi SNP chip, the ratios were also steady and close to those of the RI line. Finally, in the case of sexual chromosome Z, the ratio was 7.37 SNP.Mb^− 1^ for the RI line and 5.05 SNP.Mb^− 1^ for the L line.

Regarding the LD0.5 SNP chip (for the RI line) and the LD0.6 SNP chip (for the L line), the ratio was 8.22 ± 0.76 SNP.Mb^− 1^ and 9.47 ± 1.18 SNP.Mb^− 1^ for macro-chromosomes, 13.64 ± 2.70 SNP.Mb^− 1^ and 13.50 ± 2.23 SNP.Mb^− 1^ for intermediate chromosomes, and 23.66 ± 9.51 SNP.Mb^− 1^ and 19.92 ± 8.94 SNP.Mb^− 1^ for micro-chromosomes. As regards sexual chromosome Z, the ratio was 2.64 SNP.Mb^− 1^ for the RI line and 1.65 SNP.Mb^− 1^ for the L line.

### Influence of the size of the reference population

The influence of the size of the reference population on imputation accuracy was studied by adding individuals from previous generations in the reference population. The study was conducted for the RI line, using both methodologies. Two different cases were considered, with the imputation of G2 and G3 generations respectively as candidate populations (Table 4). The different scenarios were studied at an equivalent SNP density of 3K SNPs, for the 3Kequi and the LD0.05 SNP chips, and at an equivalent SNP density of 10K SNPs, for the 10Kequi and the LD0.5 SNP chips. In the case of the imputation of G2, the influence of the size of the reference population was studied by comparing between 580 sires of G1 and 1027 sires of (G0 + G1) as the reference population (scenarios (B) and (C)). In the case of the imputation of G3, the influence of the size of the reference population was studied by comparing between 73 sire breeders of G2, 653 sires of (G1 + male breeders of G2) and 1100 sires of (G0 + G1 + male breeders of G2) as the reference population (scenarios (D_1_), (E) and (F)).

**Table 4:** Evolution of mean correlations between true and imputed genotypes according to the size of the reference population, with the imputation of G2 and G3 as candidate population, for the RI line

Ref. pop.	G2 Imputation				G3 Imputation					
	G1 (B)		G0G1 (C)		G2♂ (D1)		G1G2♂ (E)		G0G1G2♂(F)	
SNP chip	Corr.	SE	Corr.	SE	Corr.	SE	Corr.	SE	Corr.	SE
10Kequi	0.961	0.063	0.973	0.047	0.953	0.076	0.965	0.056	0.974	0.043
3Kequi	0.884	0.113	0.914	0.085	0.868	0.136	0.896	0.103	0.912	0.087
LD0.5	0.975	0.045	0.981	0.038	0.973	0.047	0.978	0.039	0.983	0.032
LD0.05	0.899	0.057	0.921	0.083	0.892	0.107	0.914	0.081	0.929	0.068

From scenario (B) to scenario (C), at an equivalent SNP density of 3K SNPs, the mean correlations increased from 0.884 to 0.914 for the 3Kequi SNP chip, and from 0.899 to 0.921 for the LD0.05 SNP chip. At an equivalent SNP density of 10K SNPs, the mean correlations increased from 0.961 to 0.973 for the 10Kequi SNP chip, and from 0.975 to 0.981 for the LD0.5 SNP chip. Similarly, from scenario (D_1_), to (E) and (F), the increase in imputation accuracy was significant and went from 0.868 to 0.896 to 0.912 for the 3Kequi SNP chip, from 0.953 to 0.965 to 0.974 for the 10Kequi SNP chip, from 0.892 to 0.914 to 0.929 for the LD0.05 SNP chip and from 0.973 to 0.978 to 0.983 for the LD0.5 SNP chip.

### Influence of the degree of kinship between reference population and candidate population

The influence of the degree of kinship on imputation accuracy was studied on the RI line, for both methodologies and in two different cases corresponding to the imputation of G2 and G3 (Table 5). The different scenarios were studied at an equivalent SNP density of 3K SNPs, for the 3Kequi and the LD0.05 SNP chips, and at an equivalent SNP density of 10K SNPs, for the 10Kequi and the LD0.5 SNP chips. Regarding G2 imputation, a decrease in the degree of kinship was achieved from scenario (B), with 580 sires of G1 as the reference population, to scenario (G), with 447 sires of G0 as the reference population. A gap of one generation was thereby created between the reference population and the candidate population in scenario (G). As far as G3 imputation is concerned, a decrease in the degree of kinship was achieved, starting from scenario (D_1_), with 73 male breeders of G2 as the reference population, to scenario (H), with 120 male breeders of G1 as the reference population, and scenario (I), with 132 male breeders of G0 as the reference population. A gap of one generation was created between the reference population and the candidate population in scenario (H). A gap of two generations was created in scenario (I).

**Table 5:** Evolution of the mean correlations between true and imputed genotypes according to the degree of kinship between reference population and candidate population, with the imputation of G2 and G3 as candidate population

Ref. pop.	G2 Imputation				G3 Imputation					
	G1 (B)		G0 (G)		G2♂ (D1)		G1♂ (H)		G0♂(I)	
SNP chip	Corr.	SE	Corr.	SE	Corr.	SE	Corr.	SE	Corr.	SE
10Kequi	0.961	0.063	0.952	0.072	0.953	0.076	0.936	0.098	0.940	0.089
3Kequi	0.884	0.113	0.841	0.149	0.868	0.136	0.811	0.172	0.821	0.167
LD0.5	0.975	0.045	0.973	0.046	0.973	0.047	0.965	0.057	0.967	0.051
LD0.05	0.899	0.057	0.872	0.119	0.892	0.107	0.852	0.131	0.856	0.123

Regarding G2 imputation, which goes from scenario (B) to scenario (G), mean correlations decreased from 0.884 to 0.841 for the 3Kequi SNP chip, and from 0.961 to 0.952 for the 10Kequi SNP chip. For the LD0.05 SNP chip, imputation accuracy decreased from 0.899 to 0.872, whereas for the LD0.5 SNP chip, it decreased from 0.975 to 0.973. The differences in mean correlations were therefore significant.

Regarding G3 imputation, which goes from scenario (D_1_) to scenario (H), imputation accuracy decreased from 0.868 to 0.811 for the 3Kequi chip and from 0.953 to 0.936 for the 10Kequi SNP chip. For the LD0.05 and LD0.5 SNP chips, imputation accuracy respectively decreased from 0.892 to 0.852 and from 0.973 to 0.965. The differences in mean correlations were significant. However, by further increasing the gap to two generations (scenario (I)), imputation accuracy became a little bit higher, compared to the scenarios with a gap of one generation (H). The differences in mean correlations were significant, with imputation accuracy values of 0.821 for the 3Kequi SNP chip, 0.940 for the 10Kequi SNP chip, 0.856 for the LD0.05 SNP chip, and 0.967 for the LD0.5 SNP chip.

### Influence of dams genotyping

The influence of the information regarding dams on imputation accuracy was studied on the RI line, using both methodologies and by comparing scenario (D_1_) and scenario (D_2_) (Table 6). The different scenarios were studied at an equivalent SNP density of 3K SNPs, for the 3Kequi and the LD0.05 SNP chips, and at an equivalent SNP density of 10K SNPs, for the 10Kequi and the LD0.5 SNP chips. In these scenarios, G3 was the candidate population. As far as the reference population is concerned, only the 73 male breeders of G2 were taken into account for scenario (D_1_). For scenario (D_2_), the 73 male breeders and 662 female breeders of G2 were taken into account. In addition, in scenario (D_2_), imputation was done without activating the “*turnoff_fam*” option, because of the presence of sires and dams in the reference population. This enabled FImpute to use both pedigree and haplotype diversity to achieve imputations.

**Table 6:** Evolution of the mean correlations between true and imputed genotypes depending on the presence or absence of dams in the reference population

Ref. pop.	G3 Imputation			
	G2♂ (D1)		G2 (D2)	
SNP chip	Corr.	SE	Corr.	SE
10Kequi	0.953	0.076	0.983	0.036
3Kequi	0.868	0.136	0.946	0.068
LD0.5	0.973	0.047	0.989	0.026
LD0.05	0.892	0.107	0.953	0.024

Regarding scenario (D_1_), in which dams’ genotype was not taken into consideration, imputation accuracy was 0.868 for the 3Kequi SNP chip, 0.953 for the 10Kequi SNP chip, 0.892 for the LD0.05 SNP chip and 0.973 for the LD0.5 SNP chip. When adding dams’ genotype (D_2_), imputation accuracy increased to 0.946 for the 3Kequi SNP chip, 0.983 for the 10Kequi SNP chip, 0.953 for the LD0.05 SNP chip and 0.989 for the LD0.5 SNP chip. The differences in mean correlations were significant.

## Discussion

### Influence of marker density

For both lines and for both methodologies, an increase in mean correlations was observed when the number of SNPs on the low density SNP chips was increased. It was also concluded to better imputation accuracy with the LD methodology, which required less SNPs compared to the EQ methodology (for instance 16,615 SNPs versus 19,910 SNPs) for similar correlation. An inflexion point was also noticed between 5000 SNPs and 10,000 SNPs.

These results are in line with those found in the literature [7, 27], where better imputations were achieved when the number of SNPs was increased. This greater number of SNPs on the low density SNP chips, results in an increased number of genotypes present to identify the corresponding reference haplotypes. As a consequence, the probability of randomly identifying haplotypes common to the reference and candidate populations decreases.

### Influence of LD threshold

For both lines, an increase in mean correlation was observed when the LD threshold increased. The rise in mean correlations, linked to the increase in the LD threshold, can be explained by the way the selection of SNPs was done, that is by clustering the whole set of high density SNPs according to their pairwise LD. When the LD threshold is high (0.8 for instance), the number of clusters of SNPs is also high, because few pairs of SNPs are in very strong LD with each other. A great number of SNPs are therefore present on the low density SNP chips (Fig. 4). Conversely, when the LD threshold is lower (0.5 for instance), the clusters previously formed (on the basis of a LD threshold of 0.8) may become aggregated. This in turn reduces the number of SNP clusters and, as a consequence, the number of SNPs on the low density SNP chips. However, the number of SNPs on the low density SNP chip was not proportional to the LD threshold. In addition, as previously outlined, imputation accuracy decreases when the number of SNPs on the low density SNP chips is reduced.

Finally, when observing the evolution of imputation accuracy as a function of LD threshold, the inflexion point was much less distinct than it was when observing the evolution of imputation accuracy as a function of the number of SNPs on the low density SNP chips.

### Influence of MAF of imputed SNPs

Regarding the EQ methodology, the increase in imputation accuracy related to MAF was expected [25, 26]. Correlations between true and imputed genotypes are more sensitive to false imputation for SNPs with low MAF than for SNPs with high MAF. Because of the way the chip was built, the SNPs of the 10Kequi SNP chip had mostly high MAF (Fig. 5b), whereas the SNPs of the LD0.5 SNP chip had both low and high MAF (Fig. 5d), which favored a better imputation of haplotypes with a low MAF when the LD methodology was used.

### Influence of the type of chromosome

The results showed that better imputation accuracy was obtained with the LD0.5 SNP chip than with the 10Kequi SNP chip, except for the macro-chromosomes (no significant differences) and for sexual chromosome Z, where the ranking of the SNP chips was reversed. However, the results for sexual chromosome Z proved better with the EQ methodology than with the LD methodology.

For each chromosome, and consequently for each type of chromosome, the results needed to be related to the ratio of selected SNPs on the low density SNP chips, per chromosome size (SNP.Mb^− 1^). This enabled to understand the performances of each low density SNP chip, and in turn to understand the evolution of mean correlations on each low density SNP chip.

**Fig. 7:**
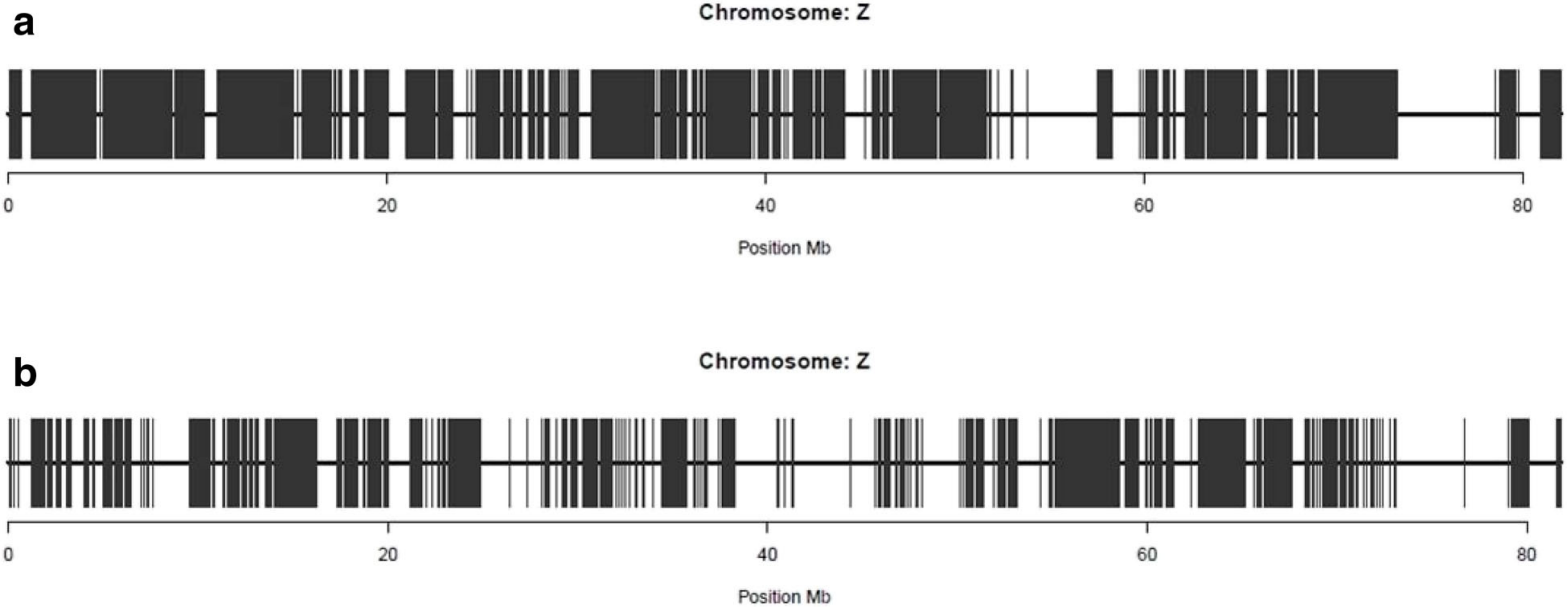
Distribution of the SNPs from the HD SNP chip on sexual chromosome Z after quality control, for the RI line (**a**) and the L line (**b**)

For the RI line (Fig. 6b) in conjunction with the 10Kequi SNP chip, the ratios were steady and consistent with the methodology used and with the size of the genome (approximately 1 Gb). Indeed, once the distance between the SNPs was defined, the ratio (SNP.Mb^− 1^) was stable and the number of SNPs on the SNP chip was proportional to the size of the chromosomes. However, in the specific case of sexual chromosome Z, the significant differences with the expected ratio of 10 SNP.Mb^− 1^ are due to the number of SNPs kept on chromosome Z after quality control. This number was 38% for the RI line and only 18% for the L line. In addition, the distribution of the SNPs kept on chromosome Z was non-homogeneous (particularly on the L line), which resulted in large intervals devoid of SNPs (Fig. 7).

Regarding the LD0.5 SNP chip (for the RI line) and the LD0.6 SNP chip (for the L line), the ratio varied depending on the type of chromosome, and the variation was higher than the variation observed with the equidistance based approach.

With the LD methodology, the specific structure of the LD in layer chickens was taken into account and the number of SNPs on each chromosome was not proportional to the size of the chromosomes. According to Robert et al. [18], for a fixed LD threshold, the extent of LD is higher on macro-chromosomes than it is on micro-chromosomes. Because of this high extent of LD on macro-chromosomes, few SNPs are needed to cover macro-chromosomes. Comparatively, the lower extent of LD on micro-chromosomes results in more SNPs necessary to cover micro-chromosomes. The LD methodology therefore enables to decrease the ratio on macro-chromosomes, in order to further optimize the number of SNPs on the low density SNP chips. Conversely, it enables to increase the ratio on intermediate and micro-chromosomes, in order to further densify the number of SNPs on the low density SNP chips. In addition, the recombination process breaks the LD and creates new haplotypes that occur more frequently on micro-chromosomes than on macro-chromosomes [28]. Overall, this resulted in better imputations with the LD SNP chips than with the EQ SNP chips for all type of chromosome and for both lines.

Finally, for both lines, better imputation accuracies were obtained with the LD methodology than with the EQ methodology. In addition, higher imputation accuracies were obtained for the L line, compared to the RI line. This was expectable because of the number of SNPs retained and distributed over the genome after quality control in both lines. The number of SNPs was greater for the RI line (300,351 SNPs) than for the L line (245,669 SNPs). The L line has therefore less polymorphic markers and can be better imputed, compared to the RI line [29].

### Influence of the size of the reference population

The increase in the size of the reference population, achieved by adding individuals from previous generations, resulted in an increase in mean correlations. Indeed, increasing the size of the reference population led to an increase in the size of the library of reference haplotypes. The probability of finding haplotype fragments of the candidate in the library of reference haplotypes was therefore increased. These results are consistent with those found in the literature [10, 11, 13].

Finally, the ranking of the methodologies remained unchanged despite the increase in the size of the reference population: the LD0.05 and the LD0.5 SNP chips respectively achieved better results than the 3Kequi and the 10Kequi SNP chips.

### Influence of the degree of kinship degree between reference population and candidate population

The gap of one generation introduced in scenarios (G) and (H), as well as the gap of two generations introduced in scenario (I), led to a decrease in the degree of kinship between the reference population and the candidate population. The downward trend in imputation accuracy in scenarios including a gap of one generation (G2 and G3 imputation) was due to a decrease in the degree of kinship between reference population and candidate population. This in turn led to a decrease in the size of haplotype fragments that are common to the reference population and the candidate population. The decrease in the size of haplotype fragments can be explained by the recombination process that occurs over the generations. Selection candidates have therefore smaller haplotype fragments in common with the reference population. The probability of mistakenly identifying a haplotype fragment common to reference population and candidate population is consequently increased, which results in a lower number of good imputations. Moreover, the decrease in imputation accuracy was higher at a density of 3K SNPs than it was at a density of 10K SNPs. Indeed, in the case of G2 imputation, there was a decrease of respectively 0.043 and 0.027 for the 3Kequi and the LD0.05 SNP chips, and a decrease of respectively 0.009 and 0.002 for the 10Kequi and the LD0.5 SNP chips. Likewise, in the case of G3 imputation, there was a decrease of respectively 0.057 and 0.040 for the 3Kequi and the LD0.05 SNP chips and a decrease of respectively 0.017 and 0.008 for the 10Kequi and the LD0.5 SNP chips. The influence of the generation gap between the reference population and the candidate population was more important at a density of 3K SNPs than it was at a density of 10K SNPs. The greater decrease in imputation accuracy at a density of 3K SNPs can be explained by the fact that the SNP density was not sufficient to compensate for the loss of imputation accuracy caused by the generation gap.

Moreover, in the case of G3 imputation, which goes from scenario (D_1_) to scenario (H), one can notice that the increase in the size of the reference population did not enable to get better imputation, in spite of the generation gap. For scenario (D_1_) the reference population was comprised of only 73 sires. In scenario (H), it was comprised of 120 sires from G1. Therefore, the loss of the information brought by the direct sires was not counterbalanced by the increase in the size of the reference population.

This observation still held true when the generation gap was further increased to two generations (scenario (I)). In that case, for both methodologies and for each low density SNP chip, a significant increase in mean correlations was noticed, compared to scenario (H). This improvement in imputation accuracy was due to the increase in the size of the reference population. However, these results were still lower than the mean correlations obtained with scenario (D_1_). With 132 sires from G0 (scenario (I)) and 120 sires from G1 (scenario (H)), the increase in the size of the reference population did not counterbalance the loss of information brought by the direct sires. Consequently, a key point to get good imputation accuracy is to include the direct parents, or at least the direct sires, of the candidate population.

Finally, the ranking of the methodologies remained unchanged, despite the decrease in the degree of kinship: the LD0.05 and LD0.5 SNP chips respectively achieved better results than the 3Kequi and 10Kequi SNP chips. In addition, in the cases of G2 and G3 imputation, and regardless of the SNP density, the decrease in imputation accuracy was less important with the LD methodology than it was with the EQ methodology. LD methodology is less sensitive to the degree of kinship, since LD does not drop very quickly through generations.

### Influence of dams genotyping

The contribution of the presence of dams in the reference population led to very high imputation accuracy. Indeed, by having both the direct sire and the direct dam of a selection candidate in the reference population, paternal and maternal haplotypes of the candidate will show in the haplotypes library. This in turn increases the probability of getting the complete genotyping of the candidate. However, it is difficult to know precisely whether the increase in imputation accuracy is due to the increase in the size of the reference population or to the presence of the dams in the reference population. As previously seen, one of the key points in imputation is to include direct sires in the reference population. One can go further by saying that it is important to have both direct sires and direct dams in the reference population, in order to get good imputation.

In addition, the results for the 3Kequi and the LD0.05 SNP chips were higher than those obtained using the same low density SNP chips, but with three generations included in the reference population (F). Therefore, when the SNP density is very low, a better alternative would be to genotype both dams and sires to achieve good imputation accuracy, rather than genotyping individuals from previous generations.

Finally, once again, the ranking of the methodologies remained unchanged when the dams were included in the reference population: the LD0.05 and the LD0.5 SNP chips still respectively achieved better results than the 3Kequi and the 10Kequi SNP chips.

## Conclusions

The above studies showed that, whatever the SNP chip used, the methodology and the scenario studied, highly accurate imputations were obtained, with mean correlations higher than 0.83. These studies also highlighted two key points allowing for good imputation results. The first one, related to SNP chip factors, is the necessity to take into consideration the particular structure of the LD of chicken species. Indeed, each time the two methodologies were compared, better results were obtained with the LD methodology. In particular, when studying the type of chromosome, except for sexual Z chromosome (for both lines), better imputation accuracies were obtained with the LD methodology. More precisely, this methodology enabled to optimize the number of SNPs on macro-chromosomes and to densify the number of SNPs on intermediate and micro-chromosomes. The second key point, related to the influence of population structure, is to include the direct parents, or at least the direct sires, of the candidate population in the reference population. Indeed, it was shown that the contribution of the direct parents (or sires) was more important than the contribution of the size of the reference population. For an equivalent quantity of information, the 10K SNPs chips achieved better results than the 3K SNPs chips. However, the results proved that the loss of imputation accuracy noticed in the case of 3K SNPs (compared to the results obtained with 10K SNPs) could be largely compensated by genotyping both the dams and the sires of the candidate population. Consequently, the choice of a very low density SNP chip will have to be considered, if new technologies are implemented with a reduction of the cost of this type of SNP chip.

Finally, the objective of genetic selection is to choose the most suitable individuals for the traits studied. The results of the genomic evaluations from all the different imputations strategies will be studied, in order to determine and finalize the best strategy to implement for low density genotyping of laying hen lines.

## Abbreviations

EQ: Equidistance
HD: High density
L: Leghorn
LD: Linkage disequilibrium
MAF: Minor allele frequency
RI: Rhode Island
SNP: Single nucleotide polymorphism
